# Fitness Loss under Amino Acid Starvation in Artemisinin-Resistant *Plasmodium falciparum* Isolates from Cambodia

**DOI:** 10.1038/s41598-018-30593-5

**Published:** 2018-08-22

**Authors:** Duangkamon Bunditvorapoom, Theerarat Kochakarn, Namfon Kotanan, Charin Modchang, Krittikorn Kümpornsin, Duangkamon Loesbanluechai, Thanyaluk Krasae, Liwang Cui, Kesinee Chotivanich, Nicholas J. White, Prapon Wilairat, Olivo Miotto, Thanat Chookajorn

**Affiliations:** 1 Genomics and Evolutionary Medicine Unit (GEM), Center of Excellence in Malaria Research, Faculty of Tropical Medicine, Mahidol University, Bangkok, Thailand; 2 Division of Medical Genetics, Department of Medicine, Faculty of Medicine, Siriraj Hospital, Bangkok, Thailand; 3 Molecular Medicine Graduate Program, Faculty of Science, Mahidol University, Bangkok, Thailand; 4 Department of Biochemistry, Faculty of Science, Mahidol University, Bangkok, Thailand; 5 Department of Physics, Faculty of Science, Mahidol University, Bangkok, Thailand; 6 Laboratory Animal Science Unit, Faculty of Tropical Medicine, Mahidol University, Bangkok, Thailand; 7 Department of Entomology, Pennsylvania State University, University Park, PA USA; 8 Department of Clinical Tropical Medicine, Faculty of Tropical Medicine, Mahidol University, Bangkok, Thailand; 9 Mahidol-Oxford Tropical Medicine Research Unit, Faculty of Tropical Medicine, Mahidol University, Bangkok, Thailand; 10 Centre for Tropical Medicine and Global Health, Nuffield Department of Medicine, University of Oxford, Oxford, UK; 11 Wellcome Sanger Institute, Hinxton, UK; 12 Medical Research Council (MRC) Centre for Genomics and Global Health, University of Oxford, Oxford, UK; 13 Present Address: Wellcome Sanger Institute, Hinxton, UK

## Abstract

Artemisinin is the most rapidly effective drug for *Plasmodium falciparum* malaria treatment currently in clinical use. Emerging artemisinin-resistant parasites pose a great global health risk. At present, the level of artemisinin resistance is still relatively low with evidence pointing towards a trade-off between artemisinin resistance and fitness loss. Here we show that artemisinin-resistant *P. falciparum* isolates from Cambodia manifested fitness loss, showing fewer progenies during the intra-erythrocytic developmental cycle. The loss in fitness was exacerbated under the condition of low exogenous amino acid supply. The resistant parasites failed to undergo maturation, whereas their drug-sensitive counterparts were able to complete the erythrocytic cycle under conditions of amino acid deprivation. The artemisinin-resistant phenotype was not stable, and loss of the phenotype was associated with changes in the expression of a putative target, Exp1, a membrane glutathione transferase. Analysis of SNPs in haemoglobin processing genes revealed associations with parasite clearance times, suggesting changes in haemoglobin catabolism may contribute to artemisinin resistance. These findings on fitness and protein homeostasis could provide clues on how to contain emerging artemisinin-resistant parasites.

## Introduction

Artemisinin and its derivatives have saved millions of malaria patients’ lives by their rapidity of action^1^. Artemisinin and its derivatives are the only drugs in clinical use that can kill every intra-erythrocytic stage of human malaria parasite *Plasmodium falciparum*^1^. Global campaigns have been launched to prevent artemisinin resistance by administering artemisinin only as combination therapies and monitoring artemisinin sensitivity by measuring parasite clearance times at key sentinel sites^2^. Despite ongoing efforts, *P. falciparum* infections with delayed parasite clearance following artemisinin treatment began to emerge in Cambodia and, after ten years, have become prevalent throughout the Greater Mekong subregion^3,4^. Even though the current artemisinin combination therapies (ACTs) can still cure *P. falciparum* malaria patients, the threat from emerging artemisinin resistance cannot be ignored, particularly since resistance to chloroquine and antifolates both spread from this region to Africa, setting back malaria control and elimination programmes for decades^5,6^.

Despite unequivocal observations of delayed parasite clearance time in malaria patients, emerging artemisinin resistance presents a unique challenge since reduced drug susceptibility is largely confined to the ring stage with the more mature stages being relatively unaffected^7^. These parasites are still responsive to artemisinin but less than before^3,4,7^. Conventional antimalarial sensitivity assays are not capable of differentiating between sensitive and resistant parasites because reduced drug susceptibility is limited to a small period during the early ring stage^8,9^. Hence, available artemisinin sensitivity assays limit the drug exposure window to early ring parasites, leading to the development of Ring Survival Assay (RSA) and Trophozoite Maturation Inhibition Assay (TMI)^9,10^. Genetic linkage analysis strongly indicated that a major determinant of delayed parasite clearance by artemisinin is located on chromosome 13^11,12^. Long-term selection under artemisinin pressure identified a mutation at *kelch 13* correlating with reduced artemisinin sensitivity^13^. The gene is located within the region on chromosome 13 strongly associated with delayed clearance^11,12^. Transgenic experiments in combination with RSA further supported the role of *kelch 13* in artemisinin resistance^14,15^. However, many parasites with *kelch 13* mutations even within the propeller domain, a fan-like structure of the protein, do not present the expected delayed clearance phenotype—and vice versa^4^. There may be more to artemisinin resistance than only *kelch 13* mutations^16,17^.

Despite being in clinical use in Southeast Asia for approximately two decades, the rise in the level of artemisinin resistance has been relatively slow in comparison to chloroquine resistance and pyrimethamine resistance. It is possible that the orchestrated campaigns to promote artemisinin combination therapy (ACT) and to prevent underdosing have kept artemisinin resistance at a relatively low level. There is also evidence indicating that the development of artemisinin resistance is costly in terms of fitness, which could balance the evolutionary selection drive towards full-blown artemisinin resistance^16^. Trade-offs between artemisinin resistance and fitness are supported by the observation that prolonged culture of artemisinin-resistant strains without artemisinin exposure leads to reduction in resistance level^10^. An *in vitro* selected artemisinin-resistant strain also loses to drug-sensitive counterparts in a growth competition assay^18^. Understanding the nature of fitness trade-offs in artemisinin resistance could impact the clinical strategy to contain resistant parasites. If these parasites adopt a secondary compensatory mutation to buffer fitness loss, high resistance levels may follow^19^.

Here, we show that artemisinin-resistant field *P. falciparum* isolates suffer from fitness loss. The parasites produce fewer progenies. The reduced fitness was exacerbated when the parasites were forced to rely on haemoglobin digestion without extra amino acid supply. The artemisinin resistance phenotype was lost when the drug pressure was removed. Association of single nucleotide polymorphisms at haemoglobin processing genes and shift in clearance time following artemisinin treatment was observed.

## Result

### Fitness loss in artemisinin-resistant parasites under amino acid starvation

**Figure 1:**
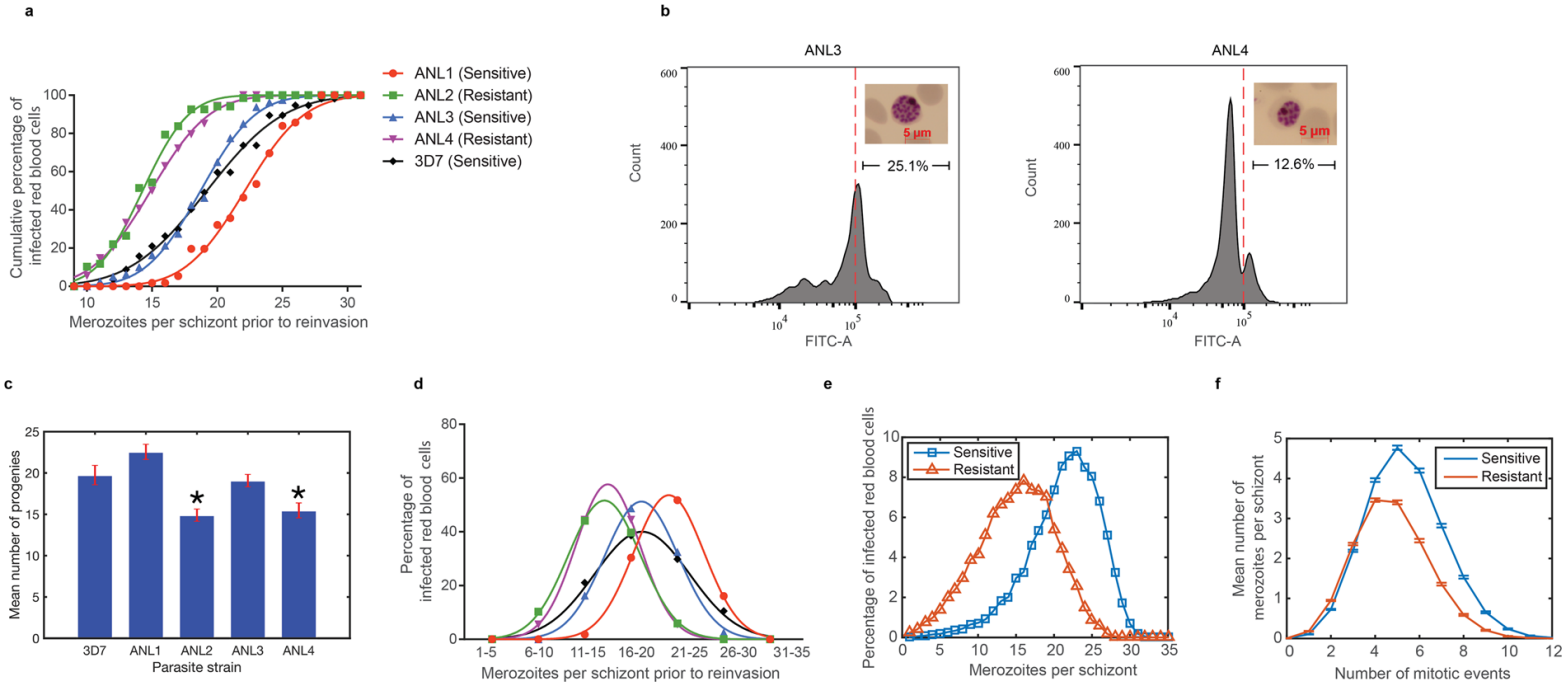
Decline in the number of progenies in artemisinin-resistant parasites. Merozoite progenies in segmented mature schizonts (~44–48 hours post invasion) were counted using Giemsa-stained thin blood smear. More than one hundred segmented mature schizonts were inspected for each strain with two microscopists randomly confirming the count. (**a**) The cumulative frequency of finding specific number of progenies was plotted for each strain (ANL2 and ANL4 for artemisinin-resistant parasites; ANL1, ANL3 and 3D7 for artemisinin-sensitive parasites). (**b**) The flow cytometry data from late-stage schizonts. The x-axis represents the florescent signal from SYBR green staining. The arbitrary line at 10^5^ is marked to show the percentage of the count (y-axis) with higher staining intensity. The insets show the average schizont-stage parasites from the strains used for flow cytometry analysis. (**c**) Mean number of progenies per schizont. *Indicates lower progeny number with statistical significance (p-value < 0.0001). ANL1, on the other hand, has more progenies than 3D7 (p-value = 0.0002). (**d**) The same dataset as in (**a**) was also presented as distribution curves. Each point represents schizont count within the bin. (**e**) Modeled distribution of the progeny numbers of drug sensitive (blue) and drug resistant (red) strains is presented at 48 hours from 10^4^ realizations. (**f**) Distribution of number of merozoites (y-axis) that underwent different number of mitotic division events at 48 hours (x-axis). The average mitosis events for sensitive and resistant strains are 5.37 and 4.76 rounds per one erythrocytic cycle, respectively. Poisson statistics was employed to determine cell division. Error bars show 95% confident intervals.

In order to study fitness trade-off, artemisinin-resistant strains (ANL2 and ANL4) from Cambodia were studied in comparison to laboratory strains and drug-sensitive isolates (ANL1 and ANL3) collected during the same period^10^. The half-life clearance time values following artemisinin treatment of ANL2 (8.55 hours) and ANL4 (8.8 hours) exceed the local median value of 6.1 hours^4^. They are consistent with the published data showing the higher IC_50_ values to artesunate of ANL2 (26 nM) and ANL4 (31.25 nM) in comparison to those of ANL1 (half-life of 5.8 hours and IC_50_ of 8.59 nM) and ANL3 (half-life of 4.6 hours and IC_50_ of 11.2 nM)^10^. An initial observation of reduced parasite growth of the resistant parasites during routine culture prompted us to determine whether it is resulted from fewer progenies. Tightly synchronized parasites were cultured, and the number of nuclei per segmented mature schizont was determined by microscopy. Indeed, the distribution curves of the progeny numbers showed a right shift, suggesting that the artemisinin-resistant parasites produced fewer progenies (the average of 21 progenies in sensitive strains as compared to 15 progenies in artemisinin-resistant ANL2 and ANL4 strains) (Fig. 1a–d). The progeny counting observation was confirmed by flow cytometry of the schizont stage parasites stained with SYBR green. The parasites were gated by forward side scatter analysis and determined DNA content by using the FITC-A channel. Fewer ANL4 parasites have the same DNA content in comparison to their artemisinin-sensitive counterpart (Fig. 1b).

To estimate the reduction in the number of mitotic divisions, we simulated cell division process using Monte Carlo algorithm (Fig. 1e). Our simulations employed an algorithm which calculates cell division rate at each time point and accepts cell division events for each cell with a probability proportional to its associated division rate [for detail see Supplementary Materials and Methods]. The simulation indicated that the difference in the number of progenies was consistent with missing approximately one round of mitosis with the average of 5.37 rounds of mitosis in the sensitive parasite and 4.76 rounds in the resistant parasite in each erythrocytic cycle (Fig. 1f). Artemisinin-resistant parasites are known to remain in the ring stage for a longer period than sensitive parasites before maturing into trophozoite, which is consistent with the reduced multiplication and poor fitness in *in vitro* selected strains^20^. It is possible that prolonged ring stage, a mechanism proposed to cause artemisinin resistance, could delay the transition toward mitotic division.

### Fitness loss is exacerbated under amino acid starvation

**Figure 2:**
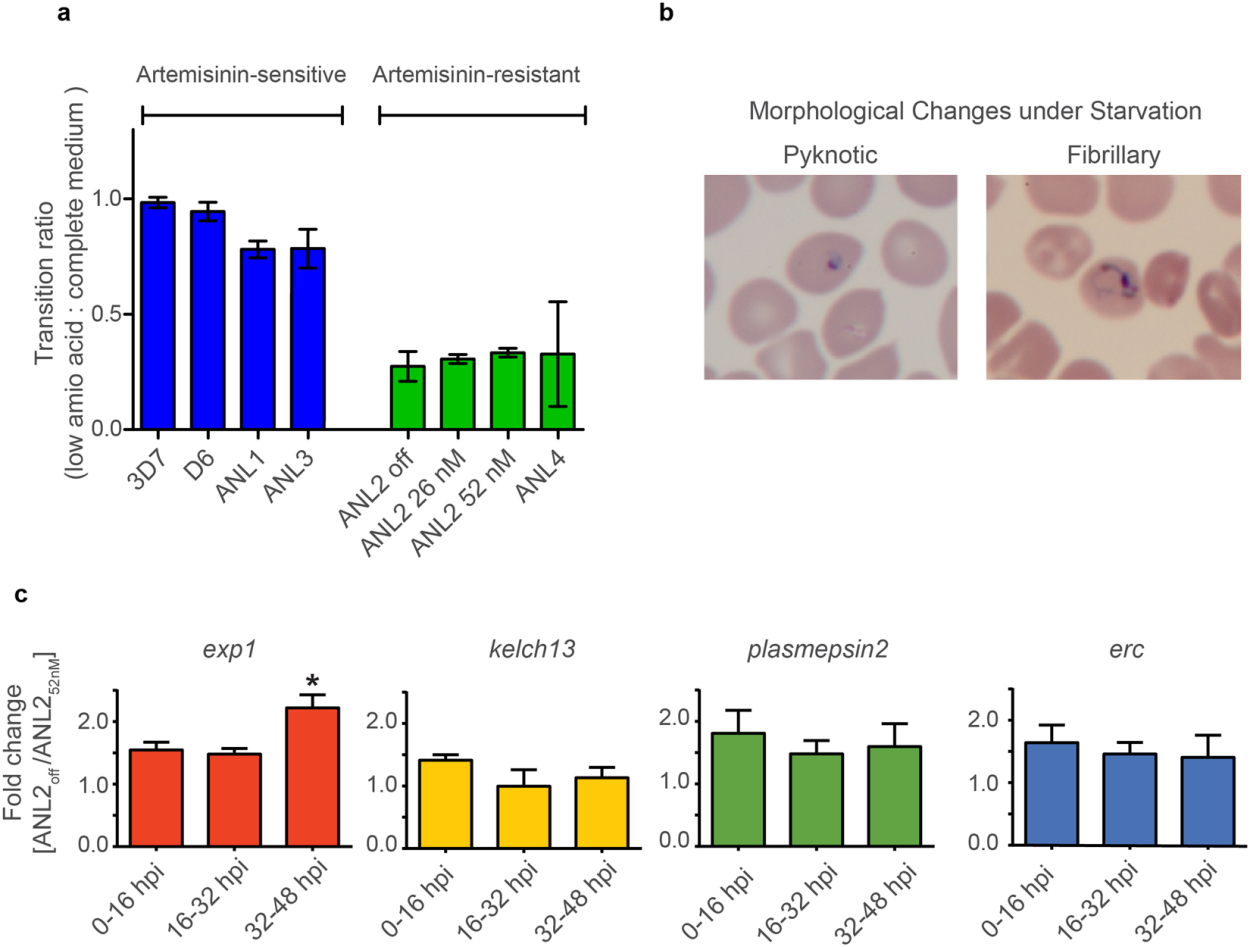
Failure to undergo transition from ring to trophozoite and schizont in artemisinin-resistant parasites under amino acid starvation. (**a**) The transition ratio represents the percentage of parasites undergoing maturation in low amino acid medium over that in complete medium in artemisinin-sensitive (blue) and artemisinin-resistant (green) parasites. ANL2_26 nM_ and ANL2_52 nM_ are artemisinin resistant parasites that are continuously under indicated drug pressure. (**b**) Morphological changes in artemisinin-resistant parasites (ANL2) under low amino acid condition showing the pyknotic form and the fibrillary form. The parasites shrank after culturing ring-stage parasites in low amino acid for 24 hours. The “pyknotic” form resembles the parasites treated with artemisinin. We noticed parasitic cytoplasm extended into small threads and named them fibrillary rings. (**c**) Changes in gene expression level in parasites losing artemisinin resistance. Fold change in the transcript level of the genes encoding EXP1, Kelch 13, Plasmepsin II, and ERC are presented as bar graph at 0–16, 16–32 and 32–48 hours post invasion (hpi). (*p-value < 0.05 with fold change greater than 2). The error bar in (**a**) and (**c**) represents standard deviation.

Artemisinin interferes with haemoglobin degradation and haemozoin formation by directly targeting released haem and/or inducing oxidative stress^21,22^. Haem is also instrumental in the activation of artemisinin at the endoperoxide bridge to become parasiticidal against *P. falciparum*^23,24^. Since loss of falcipain 2, a key haemoglobin digestive enzyme, has been linked to reduced artemisinin sensitivity, we hypothesized that decrease in haemoglobin processing might make the parasites less vulnerable to artemisinin^13,22^. However, compromised haemoglobin processing will also potentially reduce fitness, which needs to be compensated by relying more on external sources of amino acids. With this consideration, we put both artemisinin-resistant and -sensitive parasites in low amino acid medium. Loss of fitness in artemisinin-resistant strains was exacerbated in the low amino acid condition (Fig. 2a). The artemisinin-resistant strains failed to mature from ring to trophozoite whereas the sensitive strains did. This maturation failure was not observed when parasites were cultured in the standard complete culture medium. The difference in parasite maturation is presented as maturation ratio between growth in low amino acid condition versus the one in complete medium. A fraction of the resistant parasites showed morphological changes as observed by shrunken cytoplasm (pyknotic form) or extended cytoplasm (fibrillary form) (Fig. 2b).

These phenotypic changes in artemisinin resistance, though subtle, are likely to be facilitated by perturbation in gene expression. Population-wide gene expression analysis revealed small but consistent increase in those of genes encoding unfolded protein responses (UPR) in parasites from patients with delayed parasite clearance following artemisinin treatment^25^. The hallmark of UPR is the signal from the rough endoplasmic reticulum to the nucleus when protein misfolding is detected. The nucleus then responds by increasing the level of gene expression aimed to salvage imbalanced protein homeostasis in the rough endoplasmic reticulum. Recently, network analysis revealed that Exp1 is a putative target of artemisinin^26^. Exp1 was shown to be a glutathione transferase and is inhibited by dihydroartemisinin at a nanomolar range^26^. In addition, in an *in vitro*-selected parasite line showing reduced artemisinin sensitivity, there were slightly higher levels of *exp1* transcript in comparison to that of the parental line^26^. In further exploration of this issue, we maintained ANL2 in two conditions namely, with and without intermittent artemisinin exposure. ANL2 treated with 52 nM artemisinin exposure once a week had consistently high IC_50_ (14.31 ng/ml). On the other hand, the IC_50_ value of ANL2 without any artemisinin exposure for three consecutive months, now denoted ANL2_off_, fell to that of wild-type 3D7 (2.04 and 3.69 ng/ml, respectively). There was no obvious difference between the levels of *kelch 13* and *erc* (one of the UPR genes) transcripts as determined by quantitative RT-PCR (Fig. 2c). Interestingly, the level of *exp1* transcript was lower in ANL2 in comparison to ANL2_off_ especially during the schizont stage (Fig. 2c). The change was observed in every stage especially the schizonts in independent experiments (Fig. 2c).

### Association between SNPs at haemoglobin processing genes and parasite clearance time following artemisinin treatment

**Figure 3:**
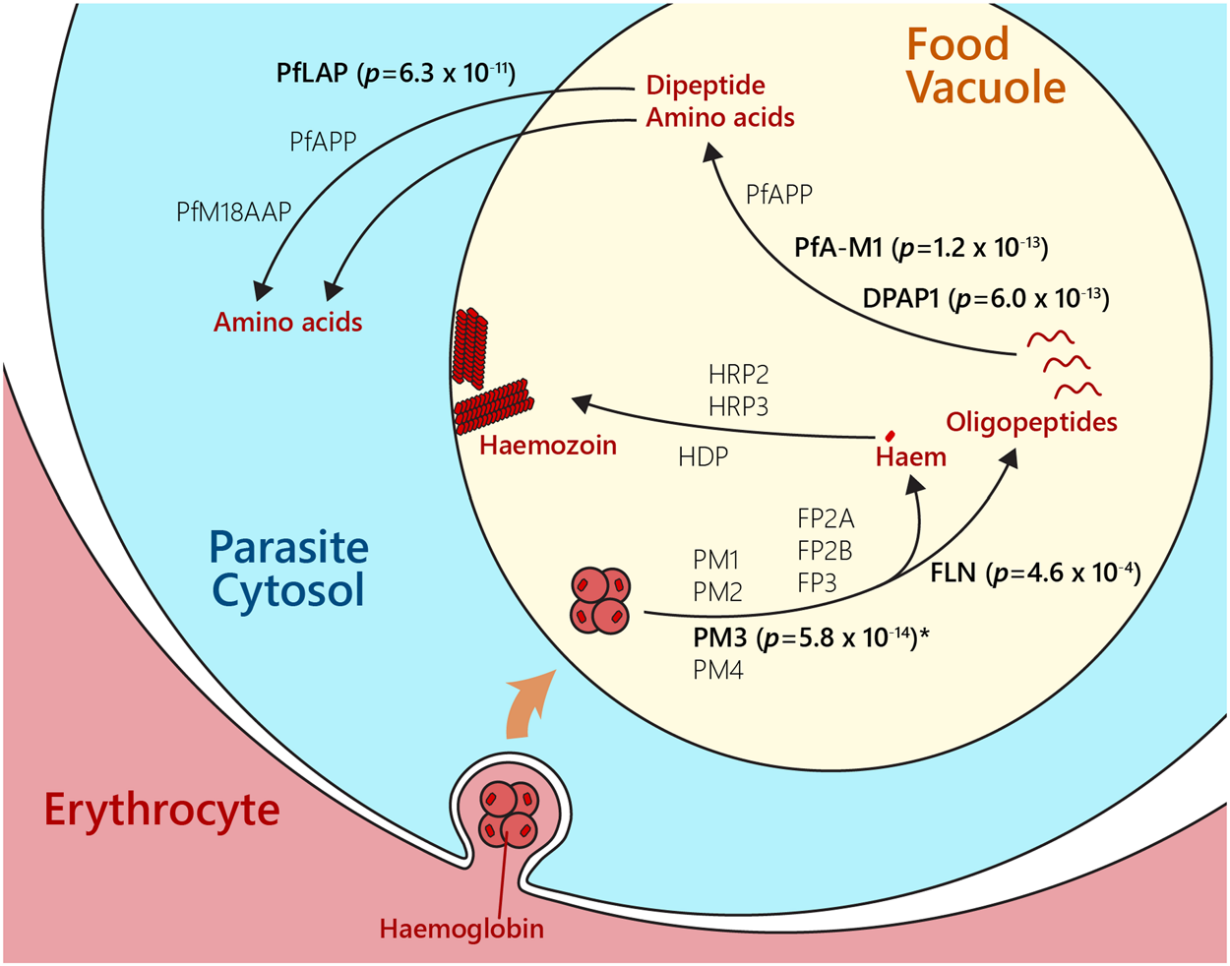
Haemoglobin-processing genes and their associations with slow and fast clearance time following artemisinin treatment. Haemoglobin from erythrocyte cytoplasm is taken up by the parasite and digested in parasite’s food vacuole using a series of proteases and peptidases. Haemoglobin processing factors having *p*-value below 0.001 are labeled in bold with the lowest *p*-value of each gene in the parentheses. The diagram shows plasmepsin (PM), falcipain (FP), falcilycin (FLN), histidine-rich protein (HRP), heme detoxification protein (HDP), dipeptidyl aminopeptidase 1 (DPAP1), M1-family alanyl aminopeptidase (PfA-M1), aminopeptidase P (PfAPP), M17-family leucyl aminopeptidase (PfLAP) and M18-family aspartyl aminopeptidase (PfM18AAP). * indicates the SNP associated with fast clearance time.

SNPs from Asian and African parasites were analysed in relation to parasite clearance times following artemisinin treatment. Parasite clearance based on quantitative parasite counts in blood smears of patients receiving artemisinin treatments was measured as the clearance half-life^27^. A total of 54,061 SNPs covering 14 chromosomes from 667 isolates were used in this study^17^. We focused on SNPs that are associated with delayed and fast parasite clearance rates. The SNPs that have been strongly associated with delayed parasite clearance are located in *ferredoxin*, *kelch 13*, *crt*, *apicoplast ribosomal protein S10 precursor* and *mdr2* as previously reported [Supplementary Table 1]^13,17^. We focused on the genes functionally linked to haemoglobin processing genes and found that the SNPs associated with these genes are associated with the change in clearance time especially for the genes encoding peptidase enzymes as shown in Table 1 and depicted in Fig. 3. One of the top SNPs is the V190I mutation at M1-family alanyl aminopeptidase (PfA-M1), listed in the top 98^th^ percentile of variations associated with delayed clearance (*p* = 1.2 × 10^−13^). It functions as a broad-spectrum aminopeptidase in the food vacuole that releases amino acid from the N-termini of oligopeptides^28,29^. Another food vacuole aminopeptidase on the list is dipeptidyl aminopeptidase 1 (DPAP1) (*p* = 6.0 × 10^−13^, 97^th^ percentile). Once cleaved, haemoglobin peptides are transported outside the food vacuole, and a group of cytosolic aminopeptidases can digest the peptides further. One of the cytosolic aminopeptidases, M17-family leucyl aminopeptidase (PfLAP), has a V600I mutation that is associated with delayed clearance (*p* = 6.3 × 10^−11^, 96^th^ percentile). The SNPs at the genes encoding haemoglobinases of the ANL parasites were previously reported^30^. These SNPs based on their association scores are not likely to be a causal mutation for artemisinin resistance by themselves, but their presence, as a whole, could be relevant to evolutionary compensation or gain of artemisinin resistance.

**Table 1:** Association between single nucleotide polymorphisms in haemoglobin-processing genes and artemisinin clearance time.

Chr	Position	Ref	Non ref	Allele freq.	Gene ID	Gene name	NS/S	Protein change	*p*-value (PLINK)	Percentile
**Association with delayed artemisinin clearance**										
8	481897	T	A	31.32%	PF3D7_0809600	C50 peptidase	NS	K5162N	3.68 × 10^−35^	99.9
13	502357	G	A	7.78%	PF3D7_1311800	PfA-M1	NS	V190I	1.22 × 10^−13^	98
11	632184	G	A	10.85%	PF3D7_1116700	DPAP1	NS	T233I	6.03 × 10^−13^	97
14	1893220	C	T	7.45%	PF3D7_1446200	PfLAP	NS	V600I	6.32 × 10^−11^	96
13	2436013	C	A	1.28%	PF3D7_1360800	Falcilysin	NS	A224D	4.63 × 10^−4^	84
**Association with fast artemisinin clearance**										
8	483714	A	G	54.06%	PF3D7_0809600	C50 peptidase	S	4557 L	7.41 × 10^−30^	100
14	298164	G	A	23.62%	PF3D7_1408100	Plasmepsin III (HAP)	NS	G233R	5.79 × 10^−14^	99.9
13	2437597	C	G	1.90%	PF3D7_1360800	Falcilysin	NS	T752S	5.25 × 10^−4^	97

Only single nucleotide polymorphisms having *p*-value < 0.001 were included in the table. Only one polymorphism per gene is shown as an example. The complete list can be found in Supplementary Table 1.

When the SNPs associated with fast parasite clearance were analysed, a member of C50 peptidase (PF3D7_0809600) is the first in the list of SNPs associated with fast clearance (Table 1 and Supplementary Table 1). C50 is a large gene family belonging to the cysteine protease clans^31^. The most well-studied member is separase which controls chromosome segregation by digestively opening the cohesion ring in several species^32^. The functions of their members are not well defined in malaria parasites^33^. Many SNPs at the gene encoding *P. falciparum* C50 cysteine protease (*PF3D7_0809600*) were found on both delayed-clearance and fast-clearance lists (Table 1 and Supplementary Table 1). A strong association between SNPs at this C50 gene and shift in clearance time indicates that its function might be relevant to artemisinin sensitivity.

Interestingly, the G223R mutation in the gene encoding Plasmepsin III (PM3) was associated with fast artemisinin clearance with the *p*-value of 5.8 × 10^−14^ (the 99.9^th^ percentile) (Table 1). This residue is located at the entry of the active site cleft, and the conversion from glycine to arginine is likely to affect substrate accessibility. Copy number variation in *pm3* was recently shown to affect piperaquine sensitivity^34,35^. These observations warrant future studies on epistatic interactions between drug resistant mutations and interplays between selective pressures from different drugs. The emergence of associated SNPs could be the effect of multiple antimalarial drugs in circulation in Cambodia and Thailand. It also does not exclude the important of the SNPs listed in Supplementary Table 1).

## Discussion

Fitness loss is a common trade-off during the evolutionary selection of antimicrobial drug resistance because antimicrobial drugs are directed against conserved critical functions. Reduced fitness has been observed in antifolate-resistant and chloroquine-resistant malaria parasites^19,36^. The evolutionary processes were traced, revealing intricate genetic interactions responsible for eventual full-blown resistance^19,36^. At present, artemisinin resistance is still emerging and spreading, which should allow preventive measures to be undertaken to thwart development of further resistance or to target the resistance mechanism directly. To date, full blown-resistance to artemisinin antimalarials has not been reported. It is worth noting that the implementation of artemisinin for more than a decade has not led to complete failure of ACT^7^. The success of artemisinin-based regimens in avoiding treatment failure thus far might reflect intrinsic properties of artemisinin in avoiding resistance.

Robustness plays an important role in drug resistance evolution^37^. By definition, robustness in evolutionary biology is an ability of the system to withstand mutational changes^38–40^. Fitness loss during the gain of drug-resistant mutations could prevent the evolutionary process to completely overcome drug pressure. Malaria parasites successfully increased robustness during antifolate resistance evolution via amplification of the gene encoding the rate-limiting step enzyme of the folate pathway^41,42^. Extra pathway flux would compensate for fitness loss during the gain of drug-resistant mutations in the folate enzyme gene downstream in the metabolic pathway^19^. Even though a well-documented report of artemisinin resistance in western Cambodia was published in 2009^3^, the parasites in Southeast Asia can still be suppressed by artemisinin-based regimens. Nevertheless, cases with longer clearance time by artemisinin have been observed in endemic areas outside western Cambodia^7^. Failure of parasites to evolutionarily reach full artemisinin resistance could be explained by the fitness loss observed in field isolates in this study and in drug-induced laboratory strains^16^. This study was performed with the parasite isolates from Cambodia. It is important to perform a similar study with more parasite isolates with reduced artemisinin sensitivity from other geographical regions since artemisinin resistance appears to be the result of convergent evolution with multiple origins^16,43^. In addition, it is necessary to verify that the loss in fitness observed under amino acid starvation is generally consistent with the rise in artemisinin resistance by expanding the study to include parasites isolates with matching clinical and laboratory phenotypes.

Two possible reasons why robustness in artemisinin resistance evolution cannot yet be achieved might be due to either its multi-target mode of action or its deleterious effect on non-protein targets. Artemisinin derivatives were shown to be cross-linked with multiple proteins in the parasites^23,44^. Since the action of artemisinin is broad and akin to an imposition of stress, changes via multiple pathways could reduce artemisinin sensitivity^16^. A matching analogy is reduction of beta-globin in thalassemia. The amount of functional globin is compromised by several mechanisms including mutations affecting globin function, transcription and RNA splicing^16^. If gain of artemisinin resistance is costly, it will be more advantageous to undergo evolutionary changes via convergent pathways^16^. The artemisinin resistance phenotype is not stable. Intermittent exposure to the drug is necessary for maintaining the phenotype. This fits well with the temporal nature of gene expression. It is consistent with the population-scale observation on the subtle transcriptomic change in parasites with reduced artemisinin susceptibility^25^.

Recent findings on mechanisms perturbing artemisinin resistance tend to involve factors controlling protein homeostasis, including UPR, ubiquitination and proteasome^25,45,46^. It is important to note that haemoglobin digestion might have the role beyond amino acid consumption. For example, haemoglobin digestion was proposed to balance the colloid-osmotic pressure to prevent the lysis of infected red blood cells^47^. The evolutionary process driving the emergence of artemisinin resistance could be progressively balancing deleterious accumulation of artemisinin-resistant mutations and fine-tuning of protein homeostasis as a fitness compensatory mechanism. The observation of fitness loss under amino acid starvation could be a phenotypic change during the evolutionary process toward fitter and more-resistant parasites which will be catastrophic to human populations living in malaria endemic zones. Efforts to eliminate these evolving parasites and to improve antimalarial development portfolio are urgently needed.

## Methods

### Malaria parasite culture

*P. falciparum* parasites were maintained based on a conventional culture method^48^. The genetic composition of the ANL parasites used in this study was previously described^30^. Low amino acid medium was prepared using RPMI 1640 Medium w/o Amino Acids, Sodium Phosphate (US Biologies, USA) and 5% freshly-prepared Albumax II (Gibco), supplemented with L-isoleucine (final concentration of 50 mg/L). Schizonts were enriched by using a Percoll gradient^49^, and ring-stage parasites were subsequently synchronized by sorbitol synchronization^50^. To test parasite development under amino acid starvation, *P. falciparum* ring-stage parasites (1% parasitemia) were cultured under low amino acid medium with conventional complete medium as a control. Parasite development was determined after 24 hours and 36 hours by Giemsa staining of standard blood smears. The experiments were performed at least in triplicates.

Flow cytometry was used to estimate the DNA content of mature schizonts^51–54^. The parasites in the schizont stage were synchronized by autoMacs Pro Separator (Miltenyl Biotec). The synchronized parasites were fixed with 1% glutaraldehyde in 1X PBS at 4 °C for 1 hour. The parasites were washed with 1X PBS and then stained with 1X SYBR green I in 1X PBS. After 1-hour incubation at room temperature, the parasites were washed twice with 1X PBS and analysed by BD FACSAria II (BD Biosciences). The events were gated by the FSC/SSC profile and by using the FITC channel to deter the signal from SYBR green staining.

### Monte Carlo simulation of cell division

The Gompertz’ growth model was used to simulate the number of progenies^55^. The number of progenies (*n*) was described by the Gompertz’ equation as follows^56^

1 $$\frac{dn}{dt}=r\,\mathrm{ln}\,(\frac{K}{n})n$$

where *K* is the carrying capacity and *r* is a constant related to the proliferative ability of the cells. The solution of the Gompertz equation can be written as (see supplementary material for detail derivation)

2 $$n(t)=K\,\exp \,[\mathrm{ln}(\frac{{n}_{0}}{K})\exp \,(-rt)]$$

where *n*_0_ = *n*(*t* = 0). Assuming that at *t* = *t*_*f*_, the average number of progenies is *n*_*f*_ and *n*_0_ = 1, substituting these in equation (2), (see supplementary material for detail derivation)

3 $$r=-\,\frac{1}{{t}_{f}}\,\mathrm{ln}\,[1-\frac{\mathrm{ln}\,({n}_{f})}{\mathrm{ln}\,(K)}]$$

Using *K* = 32,*t*_*f*_ = 48 hr, and *n*_*f*_ = 21 and 15 for drug-sensitive strain and drug-resistant strain, respectively, we obtain *r*_sensitive_ = 0.044 hr^−1^ and *r*_resistant_ = 0.032 hr^−1^.

Simulation of cell division using Monte Carlo algorithms calculated cell division rate at each time and accepted the cell division event for each cell with a probability proportional to its associated division rate. Cell division process was described by random Poisson statistics^57^. Each step of the simulation consists of: (1) calculation of the cell division rate at time *t*, $R(t)=r\,\mathrm{ln}(K/n(t))$, (2) calculation of the associated cell division probability *P*(*t*) = *R*(*t*)Δ*t* where Δ*t* is the time step in our simulation which was chosen such that *P*(*t*) is much less than one, (3) selection of a random number $a\in [0,1)$, (4) execution of a cell division event if *a* < *P*(*t*), update number of cell *n* → *n* + 1, (5) update generation of each cell *g*_*i*_ → *g*_*i*_ + 1 where *g*_*i*_ is the generation of cell *i* and (6) update of the simulation time *t* → *t* + Δ*t*. Other parameters in the simulations were set as follows: *K* = 32, *n*_0_ = 1, and Δ*t* = 0.1 hour.

### Determination of gene expression level

Ring-stage *P. falciparum* parasites were cultured and enriched twice by 5% sorbitol (Sigma)^48,50^. Parasites were collected at 0–16, 16–32 and 32–48 hours post invasion (hpi) by centrifugation at 600 × *g* for 5 minutes. RNA extraction by TRIzol Reagent (ThermoFisher Scientific) was performed according to the manufacture’s protocol. cDNA was generated by reverse transcription with Superscript III First-Strand Synthesis System using random hexamer (ThermoFisher Scientific). Quantitative PCR experiments were performed with specific primers to the genes encoding Plasmepsin II, Kelch 13, ERC and EXP1 using RBC ThermOne Real-Time PCR Premix (RBCBioscience). Real-time PCR analysis was performed using Mastercycler® ep realplex (Eppendorf) at 94 °C for 20 seconds, 60 °C for 30 seconds and 72 °C for 30 (40 cycles). Expression level was normalized as ∆Ct with the genes encoding actin and seryl-tRNA synthetase. Reactions performed without reverse transcriptase were included to monitor genomic DNA contamination.

### Genotype-phenotype association analysis

Artemisinin clearance half-life and SNP data were obtained from Tracking Resistance to Artemisinin Collaboration (TRAC) project^4,17^. Association analyses were performed using PLINK v1.07 (http://pngu.mgh.harvard.edu/purcell/plink/) and FaST-LMM v2.07^58,59^. For PLINK, *p*-value were calculated using a linear model. Heterozygous calls were excluded due to the possibility of being the result of mixed infections. Artemisinin clearance half-life was considered as quantitative trait in the analysis. Genetic similarity matrix used in FaST-LMM was calculated from SNP subset that was extracted by PLINK v1.07 using option indep-pairwise 100 10 0.3^17^.

## Electronic supplementary material

SUPPLEMENTARY INFORMATION: Solution of the Gompertz’ equation

Supplementary Dataset 1
