# Supplementary material for: Fitness Loss under Amino Acid Starvation in Artemisinin-Resistant Plasmodium falciparum Isolates from Cambodia
Source: Sci Rep. 2018 Aug 22;8:12622. doi: 10.1038/s41598-018-30593-5 (PMC6105667; doi:10.1038/s41598-018-30593-5)
Supplement: Supplementary file 1 — SUPPLEMENTARY INFORMATION: Solution of the Gompertz’ equation [file 41598_2018_30593_MOESM1_ESM.pdf]

Supplementary Material

Fitness Loss under Amino Acid Starvation in Artemisinin-Resistant *Plasmodium falciparum* Isolates from Cambodia

Duangkamon Bunditvorapoom[1,2,3,¶], Theerarat Kochakarn[1,4,¶], Namfon Kotanan[1, ¶], Charin Modchang[5], Krittikorn Kümpornsin[1,#], Duangkamon Loesbanluechai[1], Thanyaluk Krasae[6], Liwang Cui[7], Kesinee Chotivanich[8,9] , Nicholas J. White[9,10] , Prapon Wilairat[4], Olivo Miotto[9,11,12] and Thanat Chookajorn[1,*]

[1] Genomic and Evolutionary Medicine Unit (GEM), Center of Excellence in Malaria Research, Faculty of Tropical Medicine, Mahidol University, Bangkok, Thailand; [2] Division of Medical Genetics, Department of Medicine, Faculty of Medicine, Siriraj Hospital, Bangkok, Thailand; [3] Molecular Medicine Graduate Program, Faculty of Science, Mahidol University , Bangkok, Thailand; [4] Department of Biochemistry, Faculty of Science, Mahidol University, Bangkok, Thailand; [5] Department of Physics, Faculty of Science, Mahidol University, Bangkok, Thailand; [6] Laboratory Animal Science Unit, Faculty of Tropical Medicine, Mahidol University, Bangkok, Thailand; [7] Department of Entomology, Pennsylvania State University, University Park, PA, USA; [8] Department of Clinical Tropical Medicine, Faculty of Tropical Medicine, Mahidol University, Bangkok, Thailand. [9] Mahidol-Oxford Tropical Medicine Research Unit, Faculty of Tropical Medicine, Mahidol University, Bangkok, Thailand; [10] Centre for Tropical Medicine and Global Health, Nuffield Department of Medicine, University of Oxford, Oxford,

UK; [11] Wellcome Sanger Institute, Hinxton, UK; [12] Medical Research Council (MRC) Centre for Genomics and Global Health, University of Oxford, Oxford, UK.

* To whom correspondence should be addressed.

TC (thanat.cho@mahidol.edu)

¶ These authors contribute equally.

# Present address: Wellcome Sanger Institute, Hinxton, UK

SUPPLEMENTARY INFORMATION

Solution of the Gompertz' equation

From the Gompertz' equation

$$\frac{dn}{dt} = r\ln\left(\frac{K}{n}\right)n \,,$$

where n is the number of cells, K is the carrying capacity and r is a growth constant. Dividing both sides by K, we can rewrite it as:

$$\frac{d}{dt}\left(\frac{n}{K}\right) = r\left(\frac{n}{K}\right)\ln\left(\frac{K}{n}\right).$$

Let $y = n/K$, we then obtain

$$\frac{dy}{dt} = ry\ln\left(\frac{1}{y}\right).$$

We then use the separation of variables technique

$$\frac{dy}{y\ln(y)} = -rdt \,.$$

Use the substitution $z = \ln y$ and $dz = (1/y)dy$

$$\frac{dz}{z} = -rdt$$

Integrate both sides, we get

$$\int\frac{dz}{z} = -r\int dt$$

$$\ln z = -rt + C$$

where C is an integration constant. Substitute back $z = \ln y$

$$\ln\ln y = -rt + C$$

$$\ln y = e^{C}e^{-rt} = ce^{-rt}$$

$$y = \exp\left[c\exp(-rt)\right],$$

where c is another constant. So

$$\begin{aligned} n &= Ky \\ &= K\exp\left[c\exp(-rt)\right] \end{aligned}$$

Now we will determine the constant *c* by using the initial condition that when $t = 0, n = n_0$, so

$$\begin{aligned} n_0 &= K\exp\left[c\exp(0)\right] \\ &= K\exp(c) \end{aligned}$$

Hence $\exp(c) = n_0/K$ and

$$\begin{aligned} n(t) &= K\exp\left[c\exp(-rt)\right] \\ &= K\left[\exp(c)\right]^{\exp(-rt)} \\ &= K\left(\frac{n_0}{K}\right)^{\exp(-rt)} \\ &= K\left\{\exp\left[\ln\left(\frac{n_0}{K}\right)\right]\right\}^{\exp(-rt)} \\ &= K\exp\left[\ln\left(\frac{n_0}{K}\right)\exp(-rt)\right] \end{aligned}$$

To find the growth constant *r* we use the condition that $n_0 = 1$ and at $t = t_f$, $n = n_f$, therefore

$$\begin{aligned} n_f &= K\exp\left[\ln\left(\frac{1}{K}\right)\exp\left(-rt_f\right)\right] \\ &= K\exp\left[-\ln(K)\exp\left(-rt_f\right)\right] \end{aligned}$$

Take natural logarithm on both sides

$$\ln\left(\frac{n_f}{K}\right) = -\ln(K)\exp\left(-rt_f\right)$$

$$\begin{aligned} \exp\left(-rt_f\right) &= \frac{1}{\ln(K)}\ln\left(\frac{K}{n_f}\right) \\ &= \frac{1}{\ln(K)}\left[\ln(K) - \ln\left(n_f\right)\right] \\ &= 1 - \frac{\ln\left(n_f\right)}{\ln(K)} \end{aligned}$$

Hence

$$r = -\frac{1}{t_f} \ln\left[1 - \frac{\ln\left(n_f\right)}{\ln\left(K\right)}\right].$$
